# *Streptococcus suis* DivIVA Protein Is a Substrate of Ser/Thr Kinase STK and Involved in Cell Division Regulation

**DOI:** 10.3389/fcimb.2018.00085

**Published:** 2018-03-20

**Authors:** Hua Ni, Weiwei Fan, Chaolong Li, Qianqian Wu, Hongfen Hou, Dan Hu, Feng Zheng, Xuhui Zhu, Changjun Wang, Xiangrong Cao, Zhu-Qing Shao, Xiuzhen Pan

**Affiliations:** ^1^Department of Microbiology, Hua Dong Research Institute for Medicine and Biotechnics, Nanjing, China; ^2^School of Life Sciences, Nanjing Normal University, Nanjing, China; ^3^The Key Laboratory of Ecology and Biological Resources in Yarkand Oasis at Colleges and Universities Under the Department of Education of Xinjiang Uygur Autonomous Region, Kashgar University, Kashgar, China; ^4^Department of Pharmacy, Changzhou Wujin People's Hospital, Changzhou, China; ^5^State Key Laboratory of Pharmaceutical Biotechnology, School of Life Sciences, Nanjing University, Nanjing, China

**Keywords:** eukaryote-like Ser/Thr kinase, DivIVA, *Streptococcus suis* serotype 2, cell division, virulence

## Abstract

*Streptococcus suis* serotype 2 is an important swine pathogen and an emerging zoonotic agent that causes severe infections. Recent studies have reported a eukaryotic-like Ser/Thr protein kinase (STK) gene and characterized its role in the growth and virulence of different *S. suis* 2 strains. In the present study, phosphoproteomic analysis was adopted to identify substrates of the STK protein. Seven proteins that were annotated to participate in different cell processes were identified as potential substrates, which suggests the pleiotropic effects of *stk* on *S. suis* 2 by targeting multiple pathways. Among them, a protein characterized as cell division initiation protein (DivIVA) was further investigated. *In vitro* analysis demonstrated that the recombinant STK protein directly phosphorylates threonine at amino acid position 199 (Thr-199) of DivIVA. This effect could be completely abolished by the T199A mutation. To determine the specific role of DivIVA in growth and division, a *divIVA* mutant was constructed. The Δ*divIVA* strain exhibited impaired growth and division, including lower viability, enlarged cell mass, asymmetrical division caused by aberrant septum, and extremely weak pathogenicity in a mouse infection model. Collectively, our results reveal that STK regulates the cell growth and virulence of *S. suis* 2 by targeting substrates that are involved in different biological pathways. The inactivation of DivIVA leads to severe defects in cell division and strongly attenuates pathogenicity, thereby indicating its potential as a molecular drug target against *S. suis*.

## Introduction

*Streptococcus suis* (*S. suis*) is an important swine pathogen that causes a wide range of diseases, including arthritis, endocarditis, meningitis, pneumonia, and septicemia (Staats et al., 1997). *S. suis* strains have been previously classified into 35 serotypes (serotypes 1–34 and serotype 1/2) based on the antigenicity of their surface capsular polysaccharides (CPSs) (Staats et al., 1997; Gottschalk et al., 2007). However, recent genetic studies have suggested that serotypes 20, 22, 26, 32, 33, and 34 should not be attributed to *S. suis* (Hill et al., 2005; Goyette-Desjardins et al., 2014; Nomoto et al., 2015). Furthermore, among the remaining serotypes, *S. suis* 2 is considered to be the most virulent and it is frequently isolated from clinically diseased piglets (Lun et al., 2007). *S. suis* 2 caused two large outbreaks of human infection in China, which resulted in 38 deaths out of 204 reported cases and 14 deaths out of 25 cases in 2005 and 1998, respectively (Tang et al., 2006). Over the past several decades, more than 20 virulence-associated factors of *S. suis* have been identified (Vecht et al., 1991; Jacobs et al., 1996; Smith et al., 1996, 1999; de Greeff et al., 2002; Gruening et al., 2006; Feng et al., 2009; Zhang et al., 2009, 2014; Bonifait et al., 2011; Shao et al., 2011, 2013; de Buhr et al., 2014; Li et al., 2015). The expression levels of these virulence factors are affected by a series of important transcription factors, various two-component signal transduction (TCS) systems and orphan regulators (Li et al., 2008, 2011; Pan et al., 2009; Aranda et al., 2010; Fulde et al., 2011; Willenborg et al., 2011; Zheng et al., 2011; Tang et al., 2012; Wang et al., 2012).

Post-translational modifications play important roles in the modulation of protein function, signaling transduction, and various important cellular physiological processes (Michard and Doublet, 2015). Reversible protein phosphorylation caused by protein kinases and cognate phosphatases is an important post-translational modification (Ravikumar et al., 2015). In bacteria, a group of Ser/Thr kinases (STK) that is homologous to those of eukaryotic STK has been defined as eukaryote-like STK (eSTK) (Pereira et al., 2011). The bacterial eSTK is first identified from the gram-negative soil microorganism *Myxococcus xanthus* (Munoz-Dorado et al., 1991) and it has been proven to be widespread in different bacteria, including *Pseudomonas aeruginosa, Enterococcus faecalis, Staphylococcus aureus, Mycobacterium tuberculosis, Streptococcus pneumoniae, Streptococcus agalactiae*, and *Streptococcus pyogenes* (Wang et al., 1998; Av-Gay and Everett, 2000; Rajagopal et al., 2003; Echenique et al., 2004; Mougous et al., 2007; Burnside et al., 2010; Molle and Kremer, 2010; Ohlsen and Donat, 2010; Agarwal et al., 2011; Bugrysheva et al., 2011; Cameron et al., 2012; Hall et al., 2013).

Extensive studies involving phosphoproteomic profile analysis have revealed that the eSTK substrates are quite pleiotropic in nature. This property of eSTKs makes it possible to regulate numerous protein substrates for a wide range of biological processes, including transcription and translation, cell wall synthesis, and cell shape/division, sporulation, stress response, bacterial persistence, and the expression of virulence factors (Liu et al., 2011; Mata-Cabana et al., 2012; Ruggiero et al., 2012). In *S. pneumoniae*, StkP regulates cell division and morphogenesis by phosphorylating the cell initiation division protein DivIVA (Giefing et al., 2008; Nováková et al., 2010). Abolishing the phosphorylation ability of DivIVA by a threonine to alanine mutation at position 201 results in an elongated and bulged cell phenotype (Fleurie et al., 2012).

The DivIVA protein functions as a scaffold for the recruitment of other cell division-related proteins to the septum and pointed poles, and this protein is highly conserved in a wide range of gram-positive bacteria (Kaval and Halbedel, 2012; Laloux and Jacobs-Wagner, 2014). However, the biological functions of DivIVA vary among different species. For example, in *S. aureus* (Pinho and Errington, 2004), the presence or absence of the DivIVA protein does not affect cell morphology and division, whereas in *Bacillus subtilis* (Perry and Edwards, 2004), *E. faecalis* (Ramirez-Arcos et al., 2005), and *S. pneumoniae* (Nováková et al., 2010), it is essential for natural cell growth and division. In *Listeria monocytogenes* (Halbedel et al., 2012), DivIVA also influences the activity of the accessory secretion motor ATPase SecA2, which is involved in bacterial virulence factor secretion.

Recently, a STK protein was identified from *S. suis* 2. Our research group (Du et al., 2014) and other investigators (Zhu et al., 2014; Zhang et al., 2017) have revealed that the inactivation of the *stk* gene causes severe phenotype alterations, including a deficiency in cell division, decreased adhesion to host cells, greater sensitivity to H_2_O_2_, and lower pathogenicity in mice. However, the underlying molecular mechanisms remain unclear. Furthermore, while changes in the phosphorylation state after *stk* deletion in several proteins were observed (Zhang et al., 2017), whether these were directly targeted by STK and the function of these potential targets in *S. suis* 2 remain unclear. In the present study, multiple substrates of *S. suis* 2 STK were identified by phosphoproteomic and mass spectrometric analyses with a specific antibody against phosphothreonine (pThr). Furthermore, DivIVA phosphorylation by STK was validated using an *in vitro* assay. Site-directed mutagenesis showed that Thr-199 of DivIVA was essential for STK phosphorylation. To further understand the mechanism of STK in regulating cell division and growth in *S. suis*, we constructed a *divIVA*-deletion mutant and investigated its morphology and virulence alterations. Our findings indicate that STK controls cell division in *S. suis* 2 by phosphorylating the cell division initiation protein, DivIVA.

## Results

### STK deletion in *S. suis* 2 alters Thr phosphorylation levels

**Figure 1:**
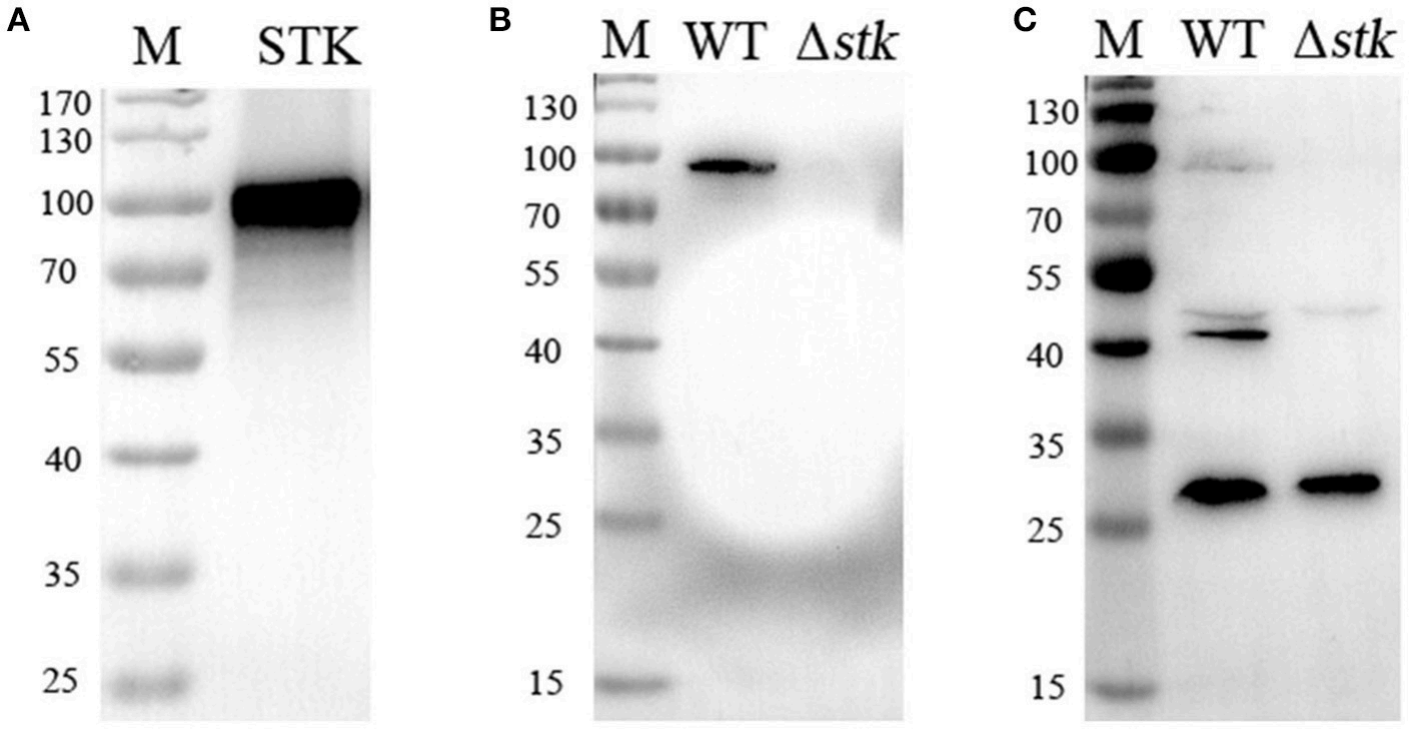
Comparison of phosphorylation levels between the WT and Δ*stk* strains. **(A)** Detection of the specificity of anti-STK polyclonal antibodies by Western blotting. **(B)** Assessment of the protein expression levels of Δ*stk*. **(C)** Thr phosphorylation levels were immunodetected in whole protein lysates of the wild-type strain and the Δ*stk* mutant strain.

To investigate the effect of the *stk* deletion on intracellular Thr phosphorylation levels, we first prepared polyclonal antibodies against STK by immunizing mice with purified recombinant STK protein. The results of the Western blot assay showed that the antibody could react specifically with the recombinant STK (Figure 1A). Then, Western blot analyses with the obtained anti-STK antibody were performed to verify the deficient expression of the STK protein in the *stk* mutant strain, and its normal expression in the WT strain (Figure 1B). Subsequently, Western blotting was performed on the total proteins extracted from the *stk* mutant and WT strain, respectively, with an anti-phosphothreonine polyclonal antibody. A comparison of the phosphorylated proteins in the cell lysates of the WT strain (05ZYH33) to the isogenic mutant (Δ*stk*) revealed at least four major phosphorylated protein bands in the WT strain, whereas only two of them were detected in the Δ*stk* strain (Figure 1C). This result preliminarily indicated that the deletion of the *stk* gene in *S. suis* partially altered the Thr phosphorylation level of its proteins.

### Genome-wide screening of putative STK substrates

**Figure 2:**
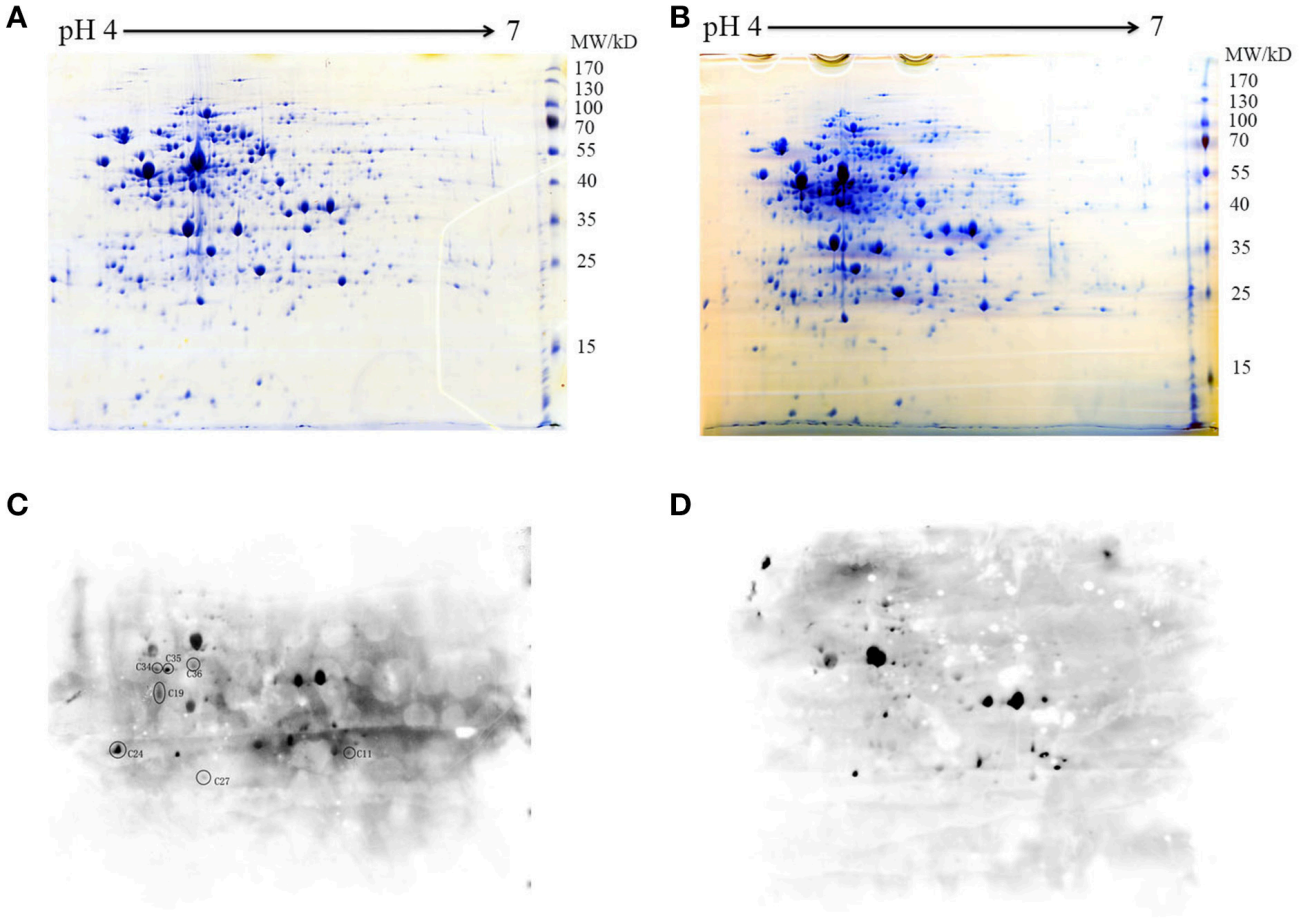
Two-dimensional map of the whole proteins of **(A)** WT and **(B)** Δ*stk*. The gels were stained with Coomassie blue **(A,B)** or electroblotted and probed with an anti-pThr antibody **(C,D)**. Protein spots C11 to C36 corresponding to phosphorylated proteins were excised and analyzed by MALDI-FTMS. Molecular weights are indicated on the right.

To further explore the potential substrates of STK, the total proteins synthesized by the WT and Δ*stk* strains were separated by two-dimensional SDS-polyacrylamide gel electrophoresis (PAGE). Each of the samples was analyzed by two gels with the same parameters. One was subjected to Coomassie blue staining (Figures 2A,B), while the other was used for detecting the Thr phosphorylation level with a specific anti-phosphothreonine polyclonal antibody (Figures 2C,D). The phosphorylation spots of the WT and Δ*stk* strains were compared, and seven WT-specific phosphorylated spots were identified, namely C11, C19, C24, C27, C34, C35, and C36 (Figure 2C). These spots at the corresponding coordinates in the Coomassie blue-stained gel were extracted for further investigation using mass spectrometry. The results annotated these proteins as ABC-type branched-chain amino acid transporter ATPase, foldase protein (PrsA), DivIVA, manganese-dependent superoxide dismutase (SodA), elongation factor Ts (EF-Ts), glucose-6-phosphate isomerase (PGI), and phosphoglycerate kinase (PGK), respectively (Table 1).

**Table 1:** Mass spectrometric identification of STK protein substrates.

**Spot**	**Accession**	**Locus**	**Protein name**	**p*I***	**Sequence coverage (%)**	**Mascot score**
C11	ABP90509	SSU05_1543	ABC-type branched-chain amino acid transport system	5.59	55	318
C19	ABP90204	SSU05_1238	Foldase protein PrsA	5.01	42	224
C24	ABP89453	SSU05_0487	Cell division initiation protein	4.54	63	328
C27	ABP90505	SSU05_1539	Manganese-dependent superoxide dismutase A	5.06	25	86
C34	ABP90945	SSU05_1979	Elongation factor Ts	4.74	56	280
C35	ABP91017	SSU05_2051	Glucose-6-phosphate isomerase	4.7	33	198
C36	ABP89127	SSU05_0157	Phosphoglycerate kinase	4.85	55	542

To evaluate the distribution and conservation of STK and its substrates among different *S. suis* strains, we surveyed the eight proteins in the 34 completely sequenced *S. suis* genomes (Table S1). The results revealed that STK and the seven potential substrates are widespread and highly conserved in all of the surveyed genomes, with each genome containing one copy of those genes. The average amino acid identity for the eight proteins from the 34 *S. suis* genomes are: 98.73 (STK), 99.25 (DivIVA), 99.47 (ATPase), 96.08 (PrsA), 99.11 (SodA), 99.21 (EF-Ts), 99.55 (PGK), and 99.47 (PGI), suggesting very low amino acid substitution among orthologous proteins.

### DivIVA is phosphorylated by STK *in vitro*

Among the seven identified proteins that show the presence/absence pattern in phosphothreonine, the C24 spot was annotated as the putative initial cell division protein DivIVA (the product of CDS *05SSU0487*). Sequence analysis revealed that the protein is composed of 229 amino acid residues, with an β-helical N-terminal domain that contains a distinct coiled coil region (3–144) and an unstable C-terminal domain (144–229) (Figure S1A). Multiple sequence alignment indicates that the amino acid sequence exhibits 67, 62, 60, and 55% amino acid sequence identity with the DivIVA of *S. pneumoniae, S. pyogenes, S. agalactiae*, and *S. mutans*, respectively (Figure S1B).

**Figure 3:**
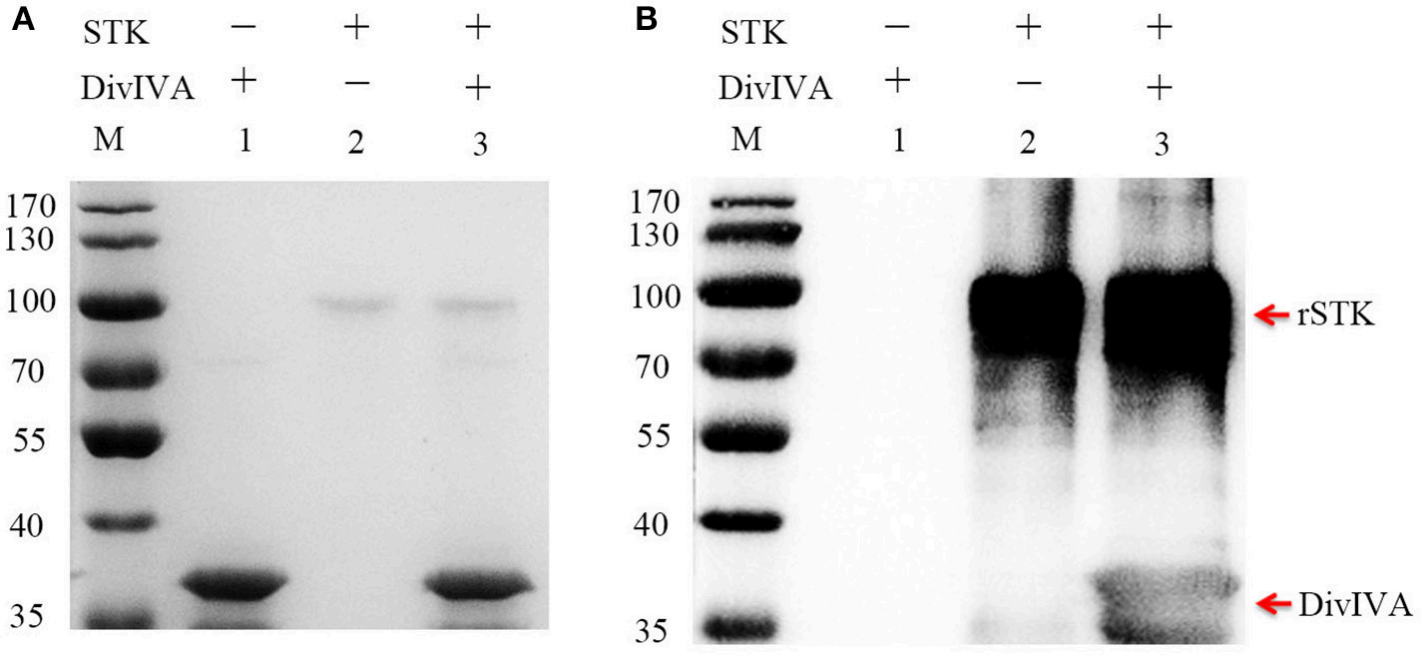
Phosphorylating of DivIVA by STK *in vitro*. **(A)** rSTK and DivIVA were incubated together, separated by SDS-PAGE, and stained with Coomassie blue. **(B)** rSTK and DivIVA were incubated together, separated by SDS-PAGE, electroblotted, and then probed with an anti-pThr antibody.

In several bacteria, DivIVA is a substrate that is directly phosphorylated by the STK protein. To test whether DivIVA is also phosphorylated by STK in *S. suis* 2, the recombinant DivIVA was expressed, purified, and subjected to *in vitro* phosphorylation by STK. First, the kinase activity of rSTK was tested by incubating with myelin basic protein (MBP), a positive control substance. The kinase assay demonstrated that rSTK is not only capable of phosphorylating MBP, but it also possesses autophosphorylation activity (Figure S2). The phosphorylation of purified rDivIVA by rSTK was then examined by immunodetection using an anti-phosphothreonine polyclonal antibody. The results show that the DivIVA protein was phosphorylated by STK, thereby suggesting that it is a genuine substrate of STK (Figure 3).

### Determination of phosphorylation sites of DivIVA

**Figure 4:**
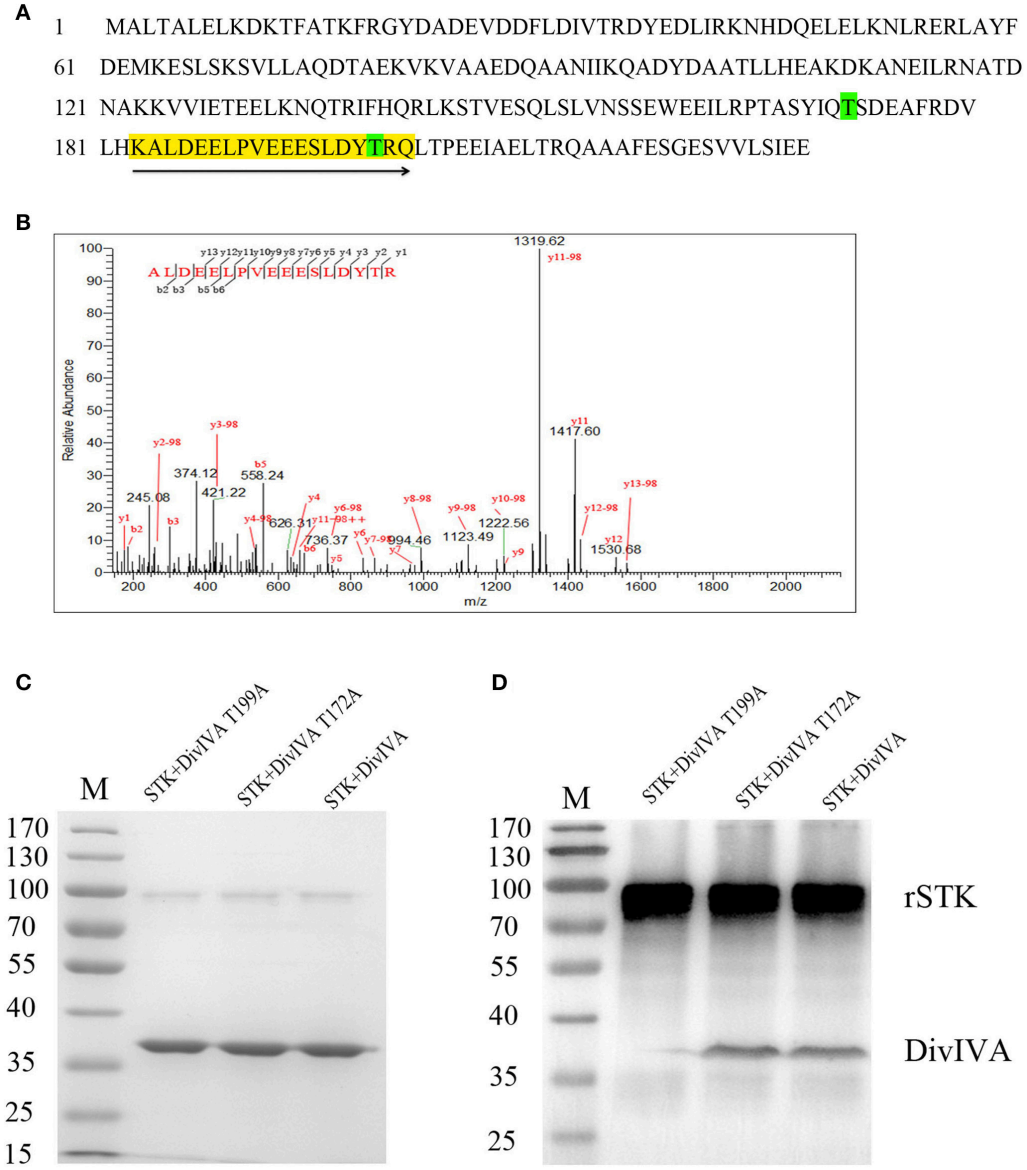
Identification of DivIVA phosphorylated sites. **(A)** Protein sequence of DivIVA: The peptide containing a phosphorylated signal is highlighted in yellow and labeled with arrows. The threonine residues introduced by mutagenesis are labeled in green. **(B)** Mass spectra showing that DivIVA is phosphorylated at threonine 199. Recombinant DivIVA was incubated with rSTK and then subjected to trypsin digestion. **(C)** rSTK incubated with mutant DivIVA proteins were separated by SDS-PAGE and then stained with Coomassie blue. **(D)** An anti-pThr antibody was used to assess the effect of mutant DivIVA proteins.

To identify the potential phosphorylation sites in DivIVA, its amino acid sequence was submitted to a kinase-specific phosphorylation site prediction online tool KinasePhos1 (http://kinasephos2.mbc.nctu.edu.tw/index.html), which predicted that the Thr-172 residue is a phosphorylation site. To further test the predictions of KinasePhos1, the *in vitro* rSTK-phosphorylated rDivIVA was subjected to trypsin digestion, followed by analysis of the digested peptides by mass spectrometry. The results showed that the DivIVA-derived peptide fragment KALDEELPVEEESLDY_P_TRQ was phosphorylated at position 17, which corresponds to T199 in the DivIVA protein sequence (Figure 4). To further validate the mass spectrometry and online prediction results, Thr-172 and Thr-199 of DivIVA were, respectively, replaced by alanine by site-directed mutagenesis. Western blot analysis revealed that the phosphorylation activity of the DivIVA-T199A protein by rSTK was completely abolished, while that of the mutant protein DivIVA-T172A did not change (Figure 4). The results suggest that STK phosphorylates DivIVA at the Thr-199 residue in *S. suis* 2.

### Phenotypic differences between WT and isogenic mutant strains

**Figure 5:**
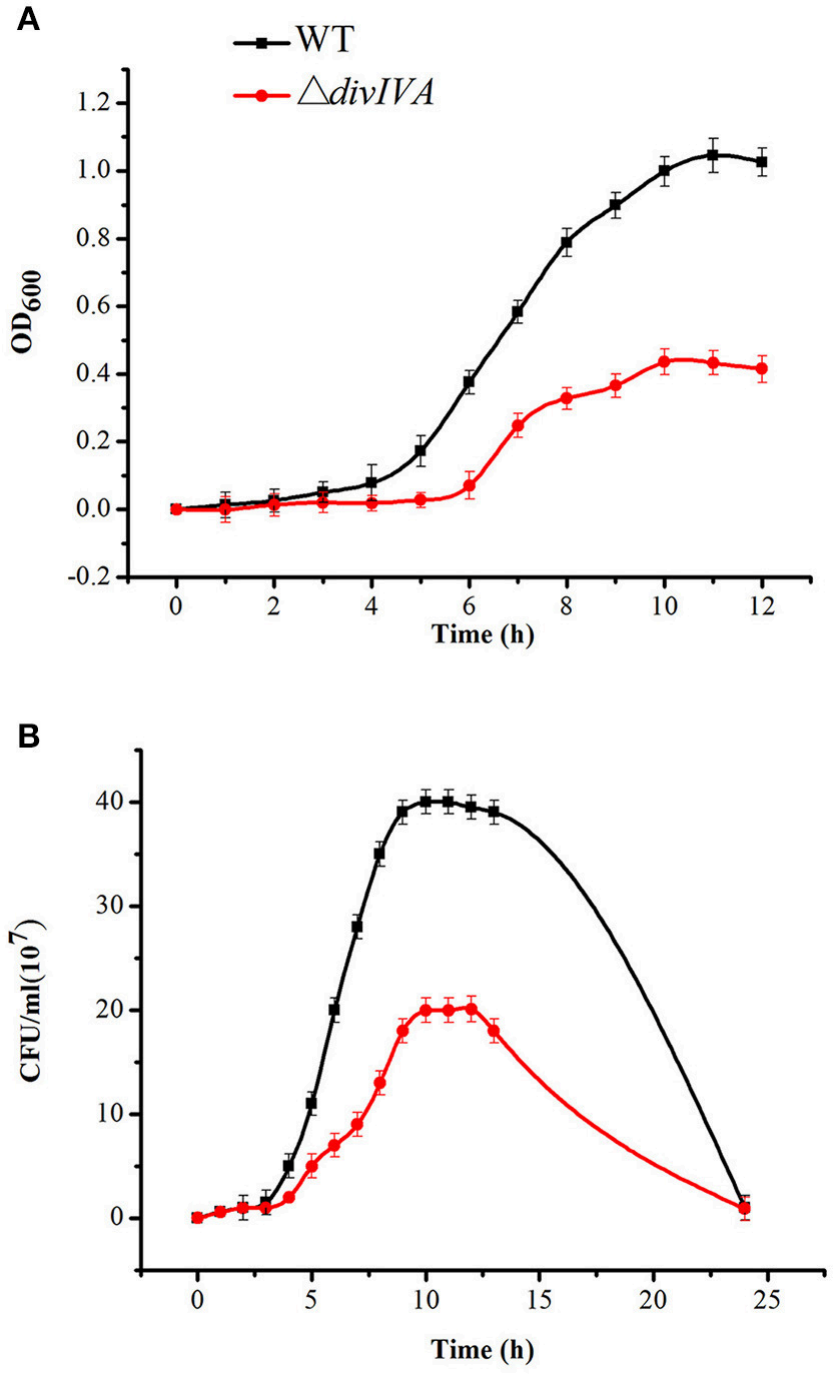
Growth characteristics of the WT and Δ*divIVA* mutant strains. **(A)** Cell density was measured spectrophotometrically at a wavelength of 600 nm, and the data were collected at the indicated time points. **(B)** Separate aliquots of the bacterial suspensions were serially diluted and plated to determine the CFU numbers per milliliter.

To investigate the role of DivIVA in the cell cycle regulation of *S. suis* 2, an isogenic *divIVA* was constructed through homologous recombination (Figure S3). Screening more than 100 Spc^r^ transformants identified one putative mutant. The double-crossover event was confirmed by combined PCR and RT-PCR analyses (Figure S3). The identified mutant strain was then renamed Δ*divIVA*. The growth of the Δ*divIVA* strain was evaluated. Compared to the wild-type strain 05ZYH33, the growth kinetics of the Δ*divIVA* strain, which was monitored by either assessing the OD_600_-values or counting the viable bacteria amounts during the initial 10–11 h of growth, was relatively slow. At that time, both the WT strain and Δ*divIVA* had already reached the post-exponential-growth phase (Figure 5).

**Figure 6:**
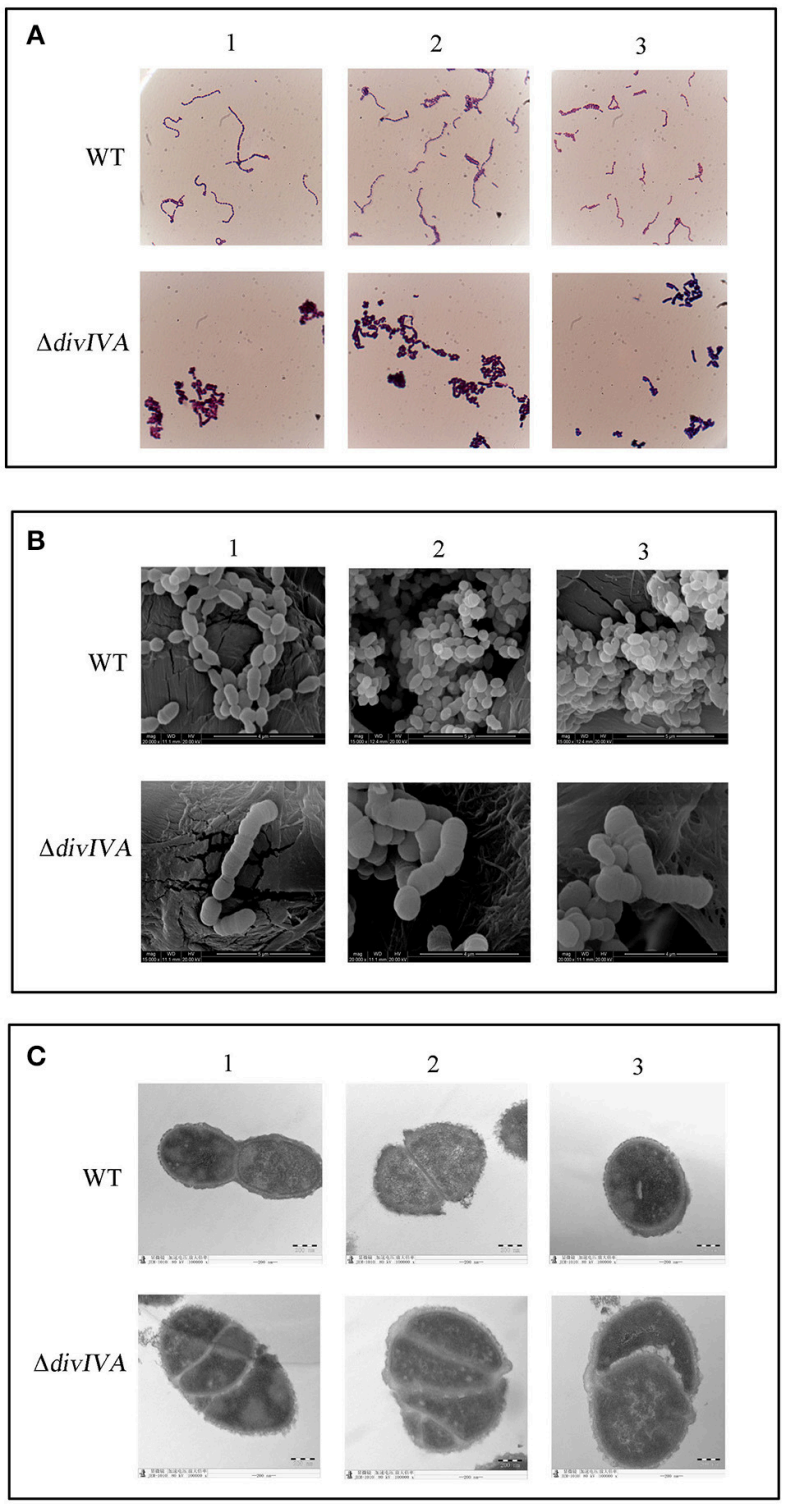
Cell morphology of the wild-type strain and the Δ*divIVA* mutant strain. **(A)** Assessment of the cellular morphology of *S. suis* using gram staining and light microscopy. **(B)** Scanning electron micrographs of the strains. **(C)** Transmission electron microscopy of the strains. The bar indicates the magnification. Each analysis is represented by three pictures captured from different fields of view.

The morphology of the WT and Δ*divIVA* strains was first examined by gram staining, which revealed that the inactivation of *divIVA* results in cell aggregation, forming a giant cell mass (Figure 6A). The SEM results show that the Δ*divIVA* strain developed into plump, sausage-shaped cells that were less ovoid than the WT cells. Some cells were unable to divide and separate, and they had lost the typical shape of *S. suis* (Figure 6B). Additionally, the results of TEM analysis reveal an irregular or disrupted cell septum in the Δ*divIVA* cells (Figure 6C). The Δ*divIVA* cells also exhibited cell division defects, including abnormal septum position and asymmetrical division. These findings indicate that DivIVA is associated with cell division and suggests that DivIVA plays a crucial role in the selection of the septum position.

### Evaluation of the pathogenicity of the *divIVA* mutant

**Figure 7:**
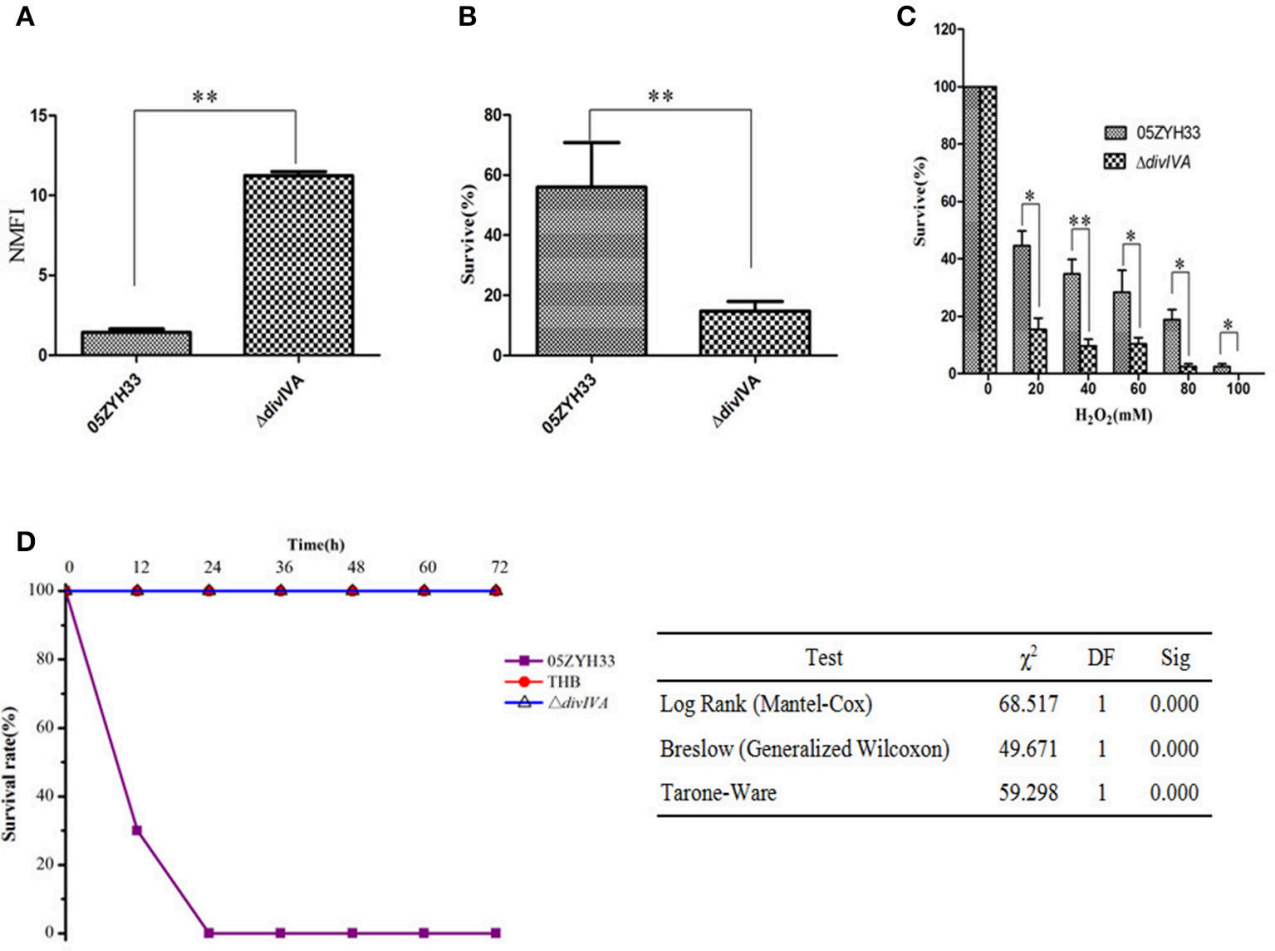
Virulence attenuation of the Δ*divIVA* mutant strain. **(A)** Evaluation the anti-phagocytotic ability of *S. suis* strains in macrophage RAW264.7 cells. **(B)** The Δ*divIVA* strain showing decreased resistance to phagocytosis and killing by neutrophils. The 10^6^ CFU of the wild-type and mutant strains was incubated with PMNs at a bacteria-to-cell ratio of 10:1. The cells were lysed after 1 h of incubation, and the survival percentage of each strain was calculated as follows: (CFU_PMN+_/CFU_PMN_) × 100%. The data are expressed as the mean and standard deviation of three independent experiments (^**^P < 0.01). **(C)** H_2_O_2_ survival test of the WT and the Δ*divIVA* mutant. *S. suis* 2 cultures at the mid-exponential phase (10^6^ CFU) were incubated with increasing concentrations of H_2_O_2_ at room temperature for 15 min, and viable counts were monitored. The assay was performed in triplicate. A statistically significant killing effect of H_2_O_2_ on the WT05ZYH33 and Δ*divIVA* mutant was observed (^*^P < 0.05; ^**^P < 0.01). **(D)** Survival curves of mice infected with *S. suis* 2 strains. Four-week-old BALB/c mice were challenged intraperitoneally with 10^8^ CFU bacteria, and the survival time was monitored. Kaplan-Meier survival analysis was performed with three different tests to evaluate the significant difference of the survival rates among the three groups.

To investigate the differences between the WT and Δ*divIVA* mutant strains during the infection of host cells, we measured their anti-phagocytosis ability against mouse macrophagocytes. Fluorescently labeled WT and Δ*divIVA* mutant strains were co-cultured with RAW264.7 cells, and then fluorescence signals in the cells were measured. The NMFI of the cells incubated with Δ*divIVA* reached 11.24 ± 0.251, which is approximately 8-fold higher than the cells incubated with WT only (1.43 ± 0.215, Figure 7A). These findings indicate that the anti-phagocytosis ability of the Δ*divIVA* strain in mouse macrophagocytes significantly decreased i*n vitro*. Furthermore, in the polymorphonuclear cell (PMN)-mediated killing assays, the Δ*divIVA* showed a lower survival rate (14.76 ± 3.17%) compared to the WT (55.96 ± 14.89%) (*P* < 0.01) (Figure 7B). These results indicate that Δ*divIVA* is more sensitive to phagocytosis and killing by PMNs. The oxidative stress test showed that the Δ*divIVA* strain is more sensitive to H_2_O_2_ than the WT strain (Figure 7C).

Mouse infection assays were further adopted to address the contribution of the *divIVA* gene to bacterial pathogenicity. The results demonstrate that all of the mice infected with the Δ*divIVA* mutant had no detectable clinical symptoms 2 days post-infection and at later time points (Figure 7D). The Δ*divIVA* mutant may possibly be avirulent in the animal model, which coincides with the findings of the cell line-based experiments. Thus, we concluded that the inactivation of DivIVA significantly attenuates the pathogenicity of *S. suis*, suggesting that the protein may be potentially used as an antibacterial target.

## Discussion

### *S. suis* 2 STK substrates are involved in different biological processes of the bacterium

eSTK regulates various cellular processes in bacteria, including cell growth and division, metabolism, stress response, and virulence. The results from the present and two previous studies (Zhu et al., 2014; Zhang et al., 2017) suggest that numerous biological activities of the *stk* mutants of *S. suis* 2 are also affected to different extents such as delayed growth rate, abnormal cell division, sensitivity to oxidative stress, and attenuated virulence. However, the underlining mechanisms of these phenotypic changes have not been well-elucidated. By performing a comparative phosphoproteomic analysis of Δ*stk* and the WT strains, we detected seven potential substrate proteins of STK that show the presence/absence of Thr phosphorylation, including PGK, PGI, SodA, EF-Ts, PrsA, DivIVA, and an amino acid transport-related protein. Literature-based function analysis revealed that the seven proteins are involved in cell division, oxidative stress, glucose metabolism, translation regulation, and protein transport process, thereby suggesting that the STK in *S. suis* 2 is equipped with a complex regulatory mechanism.

PGI catalyzes the reversible isomerization of glucose-6-phosphate (G6P) and fructose-6-phosphate (F6P) and it is involved in the regeneration of G6P molecules in the glycolytic and oxidized pentose phosphate pathway (OPPP) (Tran et al., 2009). PGK plays an important role in glycolysis and gluconeogenesis (Wang et al., 2014). The phosphorylation at threonine has been detected for PGK and PGI in *B. subtilis* (Macek et al., 2007) and *Escherichia coli* (Macek et al., 2008), suggesting a regulatory role of phosphorylation on their function. EF-Ts plays a central role in protein biosynthesis. EF-Ts regulates the abundance and stability of the EF-Tu·GTP·aminoacyl-tRNA ternary complex, which contributes to protein synthesis (Burnett et al., 2013). The threonine of EF-Ts is phosphorylated in *B. subtilis* and *L. monocytogenes* (Misra et al., 2011), suggesting that the translation process is also regulated by phosphorylation. The deficiency in threonine phosphorylation in these proteins may disrupt its function and therefore affect basic bacterial metabolism, which in turn may contribute to the delay in the growth of *stk* mutant strains.

Superoxide dismutase (SOD) are metalloproteins that convert superoxide anions to molecular oxygen and hydrogen peroxide. Manganese-dependent superoxide dismutase (SodA) is a class of SOD (McMillan et al., 2004). *S. suis* 2 utilizes SodA for survival not only by scavenging ROS but also by alleviating the host autophagic responses due to ROS stimulation (Fang et al., 2015). The inactivation of a serine/threonine phosphatase (stp1) increases *S. suis* 2 resistance to oxidative stress and survival in macrophage cells (Fang et al., 2017). In the present study, the phosphorylation of SodA was abolished in the *stk* mutant. The present study showed that Δ*stk* has decreased tolerance to H_2_O_2_ and lower survival in the anti-phagocytic experiment, which is similar to that observed in a previous study (Zhu et al., 2014). Taken together, the following scenario is proposed. Phosphorylated SodA removes ROS, which contributes to *S. suis* 2 resistance to macrophage cells. *stk* mutants have unphosphorylated SodA that is unable to remove ROS in time, thereby leading to Δ*stk* tolerance to H_2_O_2_ and anti-phagocytic decline. In contrast, when *stp1* is mutated, SodA remains phosphorylated and it is more capable of removing ROS, which in turn enhances Δ*stp* anti-phagocytosis.

### *S. suis* 2 STK regulates cell division by directly phosphorylating DivIVA protein

In *S. pneumoniae* (Fleurie et al., 2012) and *S. agalactiae* (Rajagopal et al., 2003), *stk* mutants exhibit disruptions in cell shape and size. DivIVA has been identified as a conserved target of STK in these bacteria, wherein its absence or phosphorylation deficiency significantly disrupts cell morphology and division. Our study observed a decrease in the growth rate in the Δ*stk* mutants. STK deficiency also caused an increase in the chain length and blockage of daughter cell segregation. Both our study and a previous study (Zhang et al., 2017) revealed that DivIVA phosphorylation is abolished in *stk* mutants, thereby suggesting that DivIVA is a potential target of STK in *S. suis*.

To clarify whether DivIVA plays a similar role in regulating *S. suis* cell division, a DivIVA mutant was constructed in this study. The disruption of *S. suis* DivIVA significantly affected bacterial phenotypes and growth characteristics. Similar changes in the growth rate have also been observed in *E. faecalis*, in which the turbidity and colony count measurements of the *divIVA* gene mutant strain were significantly decreased (Ramirez-Arcos et al., 2005). The absence of DivIVA in *S. suis* 2 inhibited normal cell division, which resulted in abnormal cell clusters possessing an enlarged cell mass instead of typical short chains. Furthermore, the lower viability of the *divIVA* mutant might be due to improper septa segregation within numerous cells that were generated in each cluster. These findings suggest that *divIVA* is an essential gene for the growth and maintenance of normal cell morphology in *S. suis*.

Pneumococcus cells expressing non-phosphorylatable DivIVA-T201A possess an elongated shape with a polar bulge and aberrant spatial organization of nascent peptidoglycan (Fleurie et al., 2012). DivIVA is also a key phosphorylation substrate of STK in *S. agalactiae* (Silvestroni et al., 2009), in which DivIVA reversible phosphorylation is important for normal cell division. In our study, the direct phosphorylation of DivIVA by STK was clearly detected in an *in vitro* experiment. Furthermore, we also determined that STK phosphorylates DivIVA at the Thr199 residue. The determination of the phosphorylation sites provides an important basis for further investigation on DivIVA in *S. suis* 2. In summary, our findings provide strong evidence that DivIVA is a direct substrate of STK in *S. suis* 2 that is involved in normal cell division.

### A superposition effect involving multiple pathways contributes to virulence attenuation in *stk* mutants

Apart from affecting cell morphology and division, several studies have described the involvement of *stk* in regulating bacterial virulence. In the present study and other previous investigations (Zhang et al., 2017), the attenuation of virulence was also observed in various *S. suis* 2 strains in different mouse models. The identification of various STK targets and direct evaluation of the function of DivIVA suggest that the attenuation of virulence in the *stk* mutant of *S. suis* 2 involves a superposition that is due to the disruption of the normal functions of target proteins from multiple pathways. The inactivation of each of the proteins may contribute to the observed decrease in virulence. For example, as detected in this study, the deletion of the single target gene *divIVA* in *S. suis* 2 significantly delayed cell growth, affected cell division, and attenuated virulence. Such attenuation of virulence in Δ*divIVA* is not surprising because the normal growth of bacterial cells was significantly delayed.

Similarly, three other proteins, including PGK, PGI, and EF-Ts, also play important roles in basic bacterial metabolism. Functional deficiencies in these proteins due to a disruption in phosphorylation in *stk* mutants may also contribute to decreased virulence. Unlike proteins involved in basic metabolism, SodA is a STK substrate protein that directly affects the survival of bacteria in the host environment. A recent study showed that SodA plays a role in the anti-autophagic response by scavenging ROS in infected macrophages (Fang et al., 2015). Our results suggest that the unphosphorylated state of the *stk* mutants may affect their normal function and ultimately contribute to decreased virulence. Taken together, we concluded that *stk* affects *S. suis* 2 virulence by directly modulating the phosphorylation state of some virulence factors, such as SodA. Meanwhile, the phosphorylation deficiency of STK substrates that are involved in basic cell metabolism and division may also contribute to a decrease in *S. suis* 2 pathogenicity.

## Materials and methods

### Bacterial strains, plasmids, cells, and growth conditions

The bacterial strains and plasmids used in the present study are listed in Table S2. The virulent strain *S. suis* 05ZYH33 (wild-type, WT) was isolated from an infected patient during the 2005 outbreak in Sichuan, China (Tang et al., 2006; Chen et al., 2007). The WT strain was grown in Todd-Hewitt broth (THB; Difco Laboratories, Detroit, MI, USA) liquid medium or plated on THB agar plates containing 5% (v/v) sheep blood at 37°C. Spectinomycin (100 μg/mL) was used to screen the *S. suis* mutant strain. The *E. coli* strains used in the cloning and expression of the recombinant proteins were purchased from Transgen Co. (Beijing, China). The *E. coli* DH5α, *E. coli* TOP10, and *E. coli* BL21 were maintained in Luria-Bertani (LB) broth liquid medium or plated on LB agar at 37°C. *E. coli* strain DH5α was used as host strain for cloning, while BL21 and TOP10 were used in the expression of the fusion proteins. Ampicillin or kanamycin (100 μg/ml) was added as needed.

### DNA extraction and manipulation

Genomic DNA was extracted from the WT strain using a bacterial genomic DNA extraction kit (Omega Bio-tek Co., Ohio State, USA), according to the manufacturer's instructions. The mini-preparation of recombinant plasmids and transformation of *E. coli* were performed according to standard procedures. The restriction enzymes, DNA modifying enzymes, and Taq DNA polymerases were purchased from Takara Bio Co. (Dalian, China) and used according to the manufacturer's instructions. Oligonucleotide primers were obtained from Invitrogen (Shanghai, China).

### Construction of a *divIVA* knockout mutant

A previously constructed *stk* mutant (Du et al., 2014) was used in this study. A similar method was used to construct the *divIVA* mutant strain of *S. suis* 05ZYH33. Briefly, the spectinomycin resistance (Spc^r^) gene was amplified from pSET2, and then it was inserted into a pUC18 vector (Takara, Japan) to create the recombinant plasmid pUC18-Spc. Two DNA fragments (LA and RA) flanking the *divIVA* gene were amplified from 05ZYH33 genomic DNA and cloned into pUC18-Spc to generate the knockout plasmid pUC::*divIVA*. The pUC::*divIVA* plasmid was then used for the transformation of the 05ZYH33 competent cells. The obtained transformants were confirmed by multiplex-PCR using a series of specific primers (Table 2) and reverse transcription-PCR (RT-PCR). Finally, the direct DNA sequencing of a PCR product amplified by primers Check Out1 and Check Out2 from the mutant strain was performed to confirm the successful deletion of the *divIVA* gene.

**Table 2:** Primers used for PCR amplification and detection.

**Primers**	**Sequences (5′-3′)^a^**	**Restriction enzyme**	**Amplification target**
Div-F1	GGATCCATGGCACTTACAGCATT	*Bam*HI	*divIVA* gene
Div-R1	CTCGAGTTCTTCTATTGAAAGTACTAC	*Xho*I	*divIVA* gene
STK-F1	GGATCCATGATTCAAATCGGTAAG	*Nde*I	*stk* gene
STK-R1	CTCGAGTTGTCCGCTACCTGTTG	*Hind*III	*stk* gene
Div-T172A-F1	CAACAGCAAGTTACATTCAAGCAAGTGACGAAG		Mutant *divIVA* gene
Div-T172A-F1	CTTGAATGTAACTTGCTGTTGGGCGGAGGAT		Mutant *divIVA* gene
Div-T199A-F1	AAAGTTTGGATTATACGCATCAATTGACACCAGAAG		Mutant *divIVA* gene
Div-T199A-F1	TTTCAAACCTAATATGCGTAGTTAACTGTGGTCTTC		Mutant *divIVA* gene
L1	CGAATTCGCTTTGCTAAGT TGGTTT	*Eco*RI	Upstream border of *divIVA*
L2	CGGATCCCTTTCCTCCTAAGTTTTAAC	*Bam*HI	Upstream border of *divIVA*
Spc1	GGATCCGTTCGTGAATACATGTTATA	*Bam*HI	SpcR gene
Spc2	GTCGACGTTTTCTAAAATCTGAT	*Sal*I	SpcR gene
R1	CGTCGACATTTTAAGCGAGTAGGAG	*Sal*I	Downstream border of *divIVA*
R2	GCATGCAGACTTGCTCAATAGGA	*Sph*1	Downstream border of *divIVA*
Check In1	GACGCAGATGAAGTTGATGACTT		Internal region of *divIVA*
Check In2	TGAATGTAACTTGCTGTTGGGC		Internal region of *divIVA*
Check Out1	ATATGTCGGAGCAACAAGCAAGAC		For combined PCR detection
Check Out2	TTCGATTTCAGCTTCTGCAAGG		For combined PCR detection

a *The underlined sequences are the restriction sites or nucleotides involved in site directed mutation*.

### Assessment of the growth characteristics and genetic stability of the mutant strains

The Δ*divIVA* was passaged more than 50 times and genetic stability was analyzed by PCR using the primers Spc1/Spc2. The WT and Δ*divIVA* strains were separately inoculated in flasks containing 100 mL Spc-free THB media and incubated at 37°C. The absorbance of the cultures was monitored at 1 h intervals using a spectrophotometer (Bio-Rad, USA) at a wavelength of 600 nm, and sterile THB media was used as the blank. Simultaneously, the cell cultures were also monitored using a viable bacterial count method.

### Microscopic evaluation of morphology

The WT and Δ*divIVA* strains were grown in THY broth and harvested at the mid-log phase, then washed twice with ddH_2_O. Each sample was fixed on glass slides by flaming. Gram staining was conducted according to the instructions provided in the gram staining kit (Jiancheng, China). The stained samples were observed under a light microscope.

Transmission electron microscope (TEM) and scanning electron microscope (SEM) observation were performed as previously described (Pan et al., 2009). For TEM evaluation, *S. suis* cells (WT and mutants) were harvested at an optical density at the wavelength of 600 nm (OD_600_) of 0.8 and fixed in 5% glutaraldehyde for 2 h, followed by washing with PBS. Post fixation with 1% osmium tetroxide in cacodylate buffer was conducted for 1 h at room temperature in the dark. Then, the cells were dehydrated for 20 min in each ethanol gradient and then embedded in Epon-812 epoxy resin. Ultra-thin sections were post-stained with uranyl acetate and lead citrate and observed in a JEM-1010 TEM (JEOL, Ltd., Tokyo, Japan) at an accelerating voltage of 100 kV.

For SEM analysis, all of the samples were grown in THY broth and harvested at the OD_600_ of 0.8. The cells were spotted on polylysine coverslips followed by washing with PBS (Fleurie et al., 2012). The cells were fixed in 0.18 M cacodylate buffer (pH 7.6) containing 2% glutaraldehyde. This was followed by dehydration across an ethanol gradient, passage in 1,1,1,3,3,3-hexamethyldisilazane (HMDS), and then air-drying. The dried samples were covered with a 10 nm-thick gold/platinum layer. Samples were then observed with a Quanta200 (FEI Co. Ltd., Oregon, USA) SEM.

### Oxidative stress assays

To compare H_2_O_2_ sensitivity between the WT strain and the Δ*divIVA* strain, the bacteria were grown in THB to a logarithmic phase (OD_600_ ≈ 0.6), and 10^6^ CFU cells were used in each oxidative stress assay. The WT and mutant cells were treated with 0, 20, 40, 60, 80, and 100 mM H_2_O_2_ and incubated at 37°C for 15 min. Survival rates were calculated using the following equation:

$$\begin{array}{l} \text{Survival rate}=\left(\text{CF}{\text{U}}_{\text{H}2\text{O}2+}/\text{CF}{\text{U}}_{\text{H}2\text{O}2-}\right)\times 100\text{\%}, \end{array}$$

by obtaining CFU counts from dilution plating after 48 h incubation.

### Assays for cell phagocytosis

For phagocytosis, murine macrophage Raw 264.7 cells were cultured in RPMI 1640 (Invitrogen, USA) supplemented with 10% fetal calf serum (FCS) and maintained at 37°C with 5% CO_2_. Confluent monolayers of Raw 264.7 cells grown in 24-well plates were, respectively, infected with the Δ*divIVA* and WT strains that were labeled by carboxyfluorescein diacetate, succinimidyl ester (CFSE) at a ratio of 100:1. The mixtures were incubated at 37°C with gentle agitation for 2 h, followed by penicillin and streptomycin treatment for 1 h in the dark. The Raw 264.7 and bacterial cells were pelleted, washed twice with PBS, and then fixed with 4% (wt/vol) paraformaldehyde for flow cytometry (BD) analysis. The anti-phagocytotic ability of bacteria was assessed based on the NMFI-values.

### Cell-killing experiments

Polymorphonuclear neutrophils (PMNs) were isolated from the heparinized venous blood of healthy human volunteers using the dextran sedimentation method as described by Chabot-Roy et al. (Chabot-Roy et al., 2006). Bacteria were opsonized in 10% normal human serum and incubated with human PMNs at a ratio of 10:1 for 1 h. The controls used in testing each strain were samples containing PMNs and heat-inactivated sera and samples lacking PMNs but containing human sera. The cells were lysed with 0.1% saponin (20 min on ice), and serial dilutions of the lysates were plated on THB agar. The colonies were counted, and the percentage of *S. suis* 2 bacteria that survived was determined as follows: (CFU_PMN+_/CFU_PMN−_) × 100% (Kobayashi et al., 2003). Each assay was performed in triplicate.

### Experimental infection of mice

Randomized groups of 10 4-week-old female BALB/c mice were challenged intraperitoneally with the WT, Δ*stk*, or Δ*divIVA* strains at a dose of 10^8^ CFU/mouse. THB was used as the negative control. A total of three groups was used, each consisting of 10 mice. The infected mice were monitored in terms of clinical symptoms for 2 weeks. Deaths were recorded and moribund animals were humanely killed. The animal experiments were carried out in accordance with the recommendations of the laboratory animal administration rules, State Scientific and Technological Commission. The protocol was approved by the Ethics Committee of Hua Dong Research Institute for Medicine and Biotechnics.

### Preparation of *S. suis* protein lysate and total protein

The WT and Δ*stk* strains were grown at 37°C in THB liquid medium until the desired optical densities was, respectively, reached. The cultures were harvested by centrifugation at 8,000 rpm for 10 min at 4°C. The *S. suis* cells were suspended in lysis buffer containing a protease inhibitor cocktail (Sigma, USA), respectively. The cells were disrupted by sonication to obtain crude extracts of the *S. suis* strains. The supernatant was collected by centrifugation at 12,000 rpm for 20 min at 4°C. The proteins were precipitated with acetone, and the total protein of the *S. suis* strains was collected by centrifugation. Protein concentrations were determined using a bicinchoninic acid (BCA) protein estimation kit (Pierce, USA). The protein samples were stored at −80°C.

### Identification of endogenous substrate by two-dimensional SDS-PAGE and western blotting

To further detect the phosphorylated STK protein in the WT and Δ*stk* strains, two-dimensional SDS-PAGE and Western blotting were performed. Initially, the protein extracts of the WT and *stk* mutant strains were, respectively, dissolved in a rehydration/sample buffer. Proteins were adsorbed onto an 11-cm Immobiline DryStrip (IPG, pH range 4–7, BioRad), and rehydrated overnight. For the first dimension, isoelectric focusing (IEF, BioRad) was performed using a voltage that was increased up to the steady state under the following conditions: S1, 300 V 30 min; S2, 700 V 30 min; S3, 1,500 V 1.5 h; S4, 9,000 V 3 h; and S5, 9,000 V 4 h. Prior to the second dimension, the strips were equilibrated for 2 × 15 min in equilibration buffer (6 M urea, 2% SDS, 30% glycerol, and 75 mM Tris-HCl pH 8.8, bromophenol blue), containing 2% DTT and 2.5% iodoacetamide, respectively. The proteins were separated in a 12.5% polyacrylamide gel without a spacer gel. Electrophoresis was performed at 5 mA per gel for 30 min and then at 10 mA per gel until the tracking dye reached the bottom of the gel. Each of the samples was analyzed by two gels with the same parameters. One of them was subjected to Coomassie blue staining, and the other one was detected by a specific anti-phosphothreonine polyclonal antibody. The phosphoproteins were detected with an anti-phosphothreonine polyclonal antibody at a dilution of 1:1,000, and a goat anti-rabbit secondary antibody HRP conjugate (Bio-Rad, USA) was used at a dilution of 1:8,000. STK was identified with a STK-mouse polyclonal antibody at a dilution of 1:1,000. Three replicates were run for each samples of each strain and then analyzed using the software Image Master Platinum 7.0 (GE Healthcare, New Jersey, USA). The normalized protein amount for each protein spot was calculated as the ratio of that spot's volume to the total spot's volume on the gel. A student's *t*-test (*P* < 0.05) and a threshold of 1.5-fold change in the expression spot were utilized in the data analysis.

### Mass spectrometry analysis of protein and database searches

TYPHOON SCANNE and ImageMaster 2D platinum 5.0 were employed to collect the protein point position coordinates and expression data. The phosphoprotein spots of the WT and Δ*stk* were compared, and the WT-specific spots were identified as possible substrates of STK. Furthermore, the spots of interest were visually confirmed by three independent readers prior to mass spectroscopy. Coomassie Blue-stained protein spots of interest were cut out of the gels and sent to the Guangzhou Fit Gene Biological Technology Co. Ltd. (Guangzhou, China) for trypsin in-gel digestion and Matrix-Assisted Laser Desorption/Ionization Time of Flight Mass Spectrometry (MALDI-TOF-MS) analysis. Protein spots with low Mascot scores were analyzed by MALDI-TOF/TOF-MS. Data from MALDI-TOF-MS and MALDI-TOF/TOF-MS analyses were used against the National Center for Biotechnology Information (NCBI) nr protein database using MASCOT (http://www.matrixscience.com), with the parameter settings of trypsin digestion, a maximum of one missed cleavage, variable modification of oxidation (M), and a peptide mass tolerance for monoisotopic data of 50 ppm. The sequences matching the peptide mass fingerprinting (PMF) data in MASCOT were downloaded. The probability score for the match, molecular weight (MW), isoelectric point (pI), number of peptide matches, and percentage of the total translated open reading frame (ORF) sequence covered by the peptides were analyzed for confident spot identification.

### Protein expression and purification

The primers used in the amplification of target DNA fragments in the present study are listed in Table 2. The coding sequence of *stk* and *divIVA* was amplified using the primers STK-F1/STK-R1 and Div-F1/Div-R1 from the 05ZYH33 genome, respectively. The PCR products of *stk* and *divIVA* were, respectively, restricted with *Nde*I/*Hind*III or *Bam*HI/*Xho*I, respectively, and then inserted into the digested pET-30a and pET-28a vector to generate the recombinant plasmids pET-*stk* and pET-*divIVA*, which were then transformed into *E. coli* TOP10 and *E. coli* BL21 (DE3) cells, respectively. When the OD_600_ of the cells reached around 0.8, the expression was induced by adding 1 mM isopropyl-β-D-thiogalactopyranoside (IPTG) (Sigma, USA) and incubating at 16°C for 12 h. His-tagged rSTK and rDivIVA were purified using Ni-NTA columns (GE Healthcare, Sweden) according to the manufacturer's recommendations. The purified protein was identified by Western blot analysis using a corresponding peroxidase-conjugated anti-polyhistidine monoclonal antibody (Sigma, USA).

### Production of antibodies against the rSTK and rDivIVA proteins

Adult female New Zealand white rabbits were used for the production of antibodies. Approximately 400 μg of rDivIVA was emulsified in an equal volume of complete Freund's adjuvant (CFA, Sigma-Aldrich, USA) and injected into the white rabbits intradermally. Rabbits were given two booster injections of 200 μg of protein with incomplete Freund's adjuvant (IFA, Sigma-Aldrich, USA) every 2 weeks. The rabbits received the final immunization consisting of 400 μg of purified rDivIVA protein without complete/incomplete Freund's adjuvant. Seven days later, the rabbits were bled, and the sera were separate and assayed for antibodies against rDivIVA. A similar approach was used in the production of the rSTK polyclonal antibodies using Balb/c mice. Western blotting was performed to assess the specificity of the polyclonal antibodies produced by two different animals.

### *In vitro* protein phosphorylation

Approximately 0.34 μg of rSTK was incubated with the substrate protein (1.0 μg−1.2 μg) in a kinase buffer containing 50 mM Tris-HCl (pH 7.5), 25 mM NaCl, 10 mM MnCl_2_, 1 mM MgCl_2_, 1 mM dithiothreitol, 0.1 mM EDTA, and 10 mM ATP for 30 min at 37°C. The reaction was stopped by adding 5 × SDS sample buffer. The proteins were separated by 10% SDS-PAGE and transferred onto a PVDF membrane. The proteins were detected with an anti-phosphothreonine polyclonal antibody. For substrate phosphorylation, MBP (Sigma, USA) was used as the positive control in testing for kinase activity.

### Determination of phosphorylation sites in DivIVA

First, the DivIVA amino acid sequence was submitted to a kinase-specific phosphorylation site prediction online tool, KinasePhos1 (http://kinasephos2.mbc.nctu.edu.tw/index.html) to predict the phosphorylated residues. Then, rDivIVA was phosphorylated by rSTK *in vitro* and then separated on a 10% SDS polyacrylamide gel. The band corresponding to rDivIVA was excised and subjected to trypsin digestion, and then the digested peptides were analyzed by mass spectrometry. Based on the results of network analysis and amino acid substitution predictions, we then conducted site-directed mutagenesis. First, PCR was performed using pET28a-*divIVA* as a template with the primer pairs DivIVA-T172A-F1/DivIVA-T172A-R1 and DivIVA-T199A-F1/DivIVA-T199A-R1 to generate pET28a-*divIVA*T172A and pET28a-*divIVA*T199A. Second, the two plasmids containing the Ala mutation at position 172 or 199 were digested by the *Dpn*I restriction enzyme and then transformed into the *E. coli* competent cells, respectively. The resulting constructs were then verified by DNA sequencing. Finally, the plasmids pET28a-*divIVA*T172A and pET28a-*divIVA*T199A were extracted from the *E. coli* cells, respectively, and then transformed into *E. coli* BL21 cells. The site-directed mutated protein was overexpressed, purified, and subjected to phosphorylation assays as earlier described earlier.

### Statistical analysis

All of the assays were performed in triplicate at least three times. Statistical analysis was performed using a student's *t*-test. Differences were considered to be statistically significant when the calculated *P*-value was < 0.05.

## Author contributions

XP, Z-QS, and XC conceived and designed the project. HN and WF performed most experiments and data analysis. CL, QW, HH, DH, FZ, XZ, and CW participated in some experiments and data analysis. XP, Z-QS, HN, and WF wrote the paper. All authors contributed to discussion of the results, reviewed the manuscript, and approved the final article.

### Conflict of interest statement

The authors declare that the research was conducted in the absence of any commercial or financial relationships that could be construed as a potential conflict of interest.

## Supplementary material

Figure S1 Sequence analysis of *S. suis* DivIVA. **(A)** Schema of *S. suis* DivIVA protein. N terminal coiled-coil DivIVA domain and C terminal unstable region, respectively. **(B)** Multiple sequence alignment of *S. suis* DivIVA with related homologous proteins at the amino acid level.

Figure S2 Kinase activity of STK *in vitro*. **(A)** After incubating rSTK with MBP as a positive substrate control, the protein was separated by SDS-PAGE and stained with Coomassie blue. **(B)** After incubating rSTK with MBP, the proteins were separated by SDS-PAGE, electroblotted, and then probed with an anti-pThr antibody.

Figure S3 Construction of an isogenic *divIVA* mutant of *S. suis* 05ZYH33. **(A)** Diagram of gene *divIVA* knockout. The *divIVA* gene represented by a thick orange arrow was replaced by the spectinomycin resistance gene, as indicated by a yellow arrow. The thin arrows indicate the primer positions used in the construction and identification of the *divIVA* knockout mutant (Δ*divIVA*). **(B)** Confirmatory PCRs of the Δ*divIVA* mutant. The primer pairs and templates used in the PCR analysis are indicated above the lanes. The WT and Δ*div* represent genome DNA of the wild-type strain 05ZYH33 and mutant strain, respectively. **(C)** Extraction of total RNAs. Total RNAs were extracted from the wild-type strain 05ZYH33 and the Δ*divIVA* mutant. **(D)** RT-PCR analysis of the *divIVA* gene transcripts. The transcripts of the *spc* and *divIVA* genes were detected using reverse transcription (RT-PCR) analysis with cDNA, cDNA reaction mixtures without reverse transcriptase (cDNA-RT), or genomic DNA (gDNA) as templates. The RT-PCR products were analyzed by electrophoresis on a 1.0% agarose gel (lanes 1, 2, and 3 represent the application using gDNA, cDNA-RT, and cDNA from the mutant Δ*divIVA* strain as template, respectively, and Spc1/Spc2 primers; lanes 4, 5, and 6 represent the application using gDNA, cDNA-RT, and cDNA from the mutant Δ*divIVA* strain as a template, respectively, and In1/In2 primers; and lanes 7, 8, and 9 represent the application using gDNA, cDNA-RT, and cDNA from the wild-type strain 05ZYH33 as a template respectively, and In1/In2 primers).

Table S1 Genomes used in this study.

Table S2 Bacterial strains and plasmids used in this study.
