# Supplementary figures and images for: Streptococcus suis DivIVA Protein Is a Substrate of Ser/Thr Kinase STK and Involved in Cell Division Regulation
Source: Front Cell Infect Microbiol. 2018 Mar 20;8:85. doi: 10.3389/fcimb.2018.00085 (PMC5869912; doi:10.3389/fcimb.2018.00085)

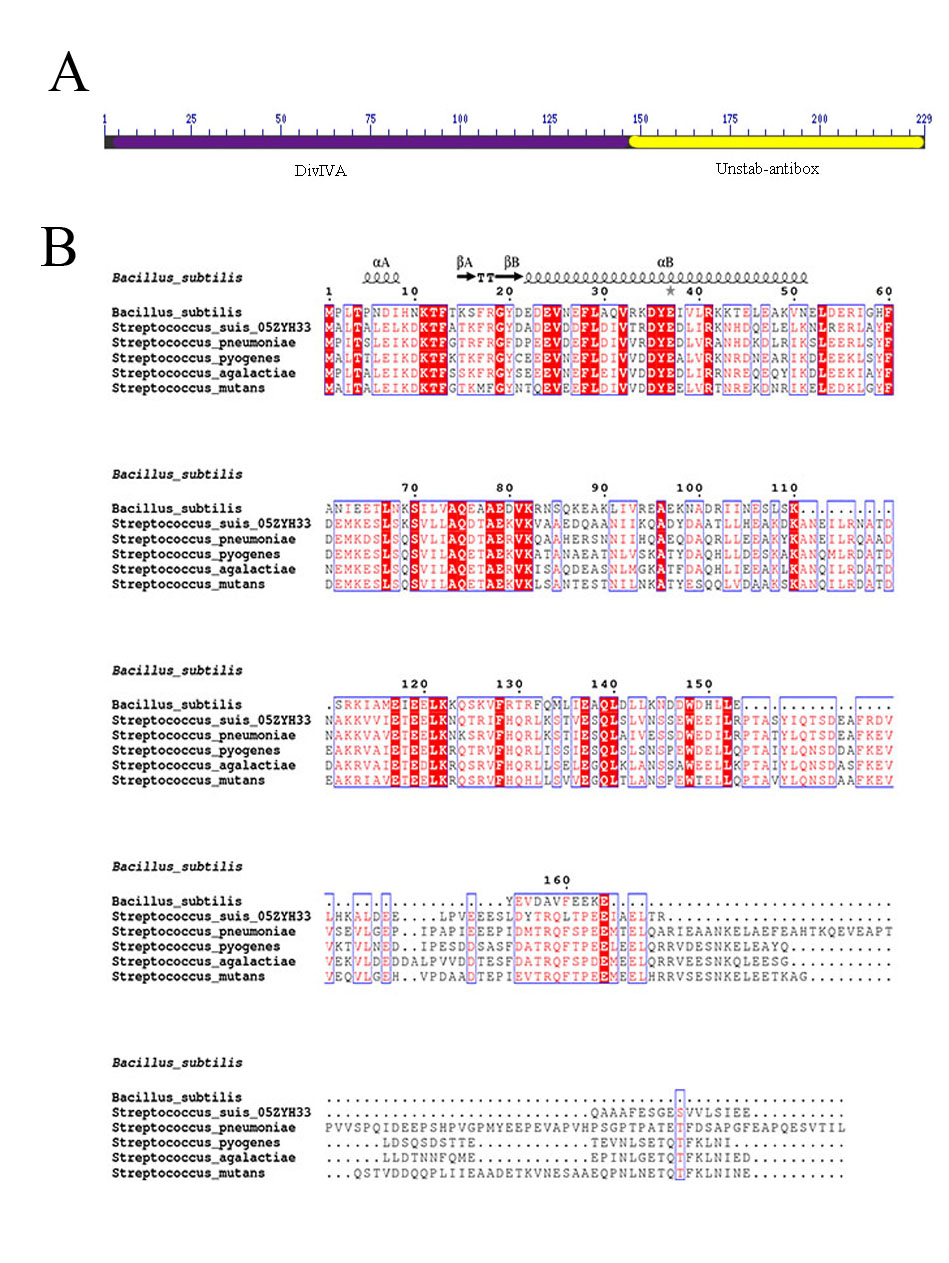

Supplement: Figure S1 — Sequence analysis of S. suis DivIVA. (A) Schema of S. suis DivIVA protein. N terminal coiled-coil DivIVA domain and C terminal unstable region, respectively. (B) Multiple sequence alignment of S. suis DivIVA with related homologous proteins at the amino acid level. [file Image1.JPEG]

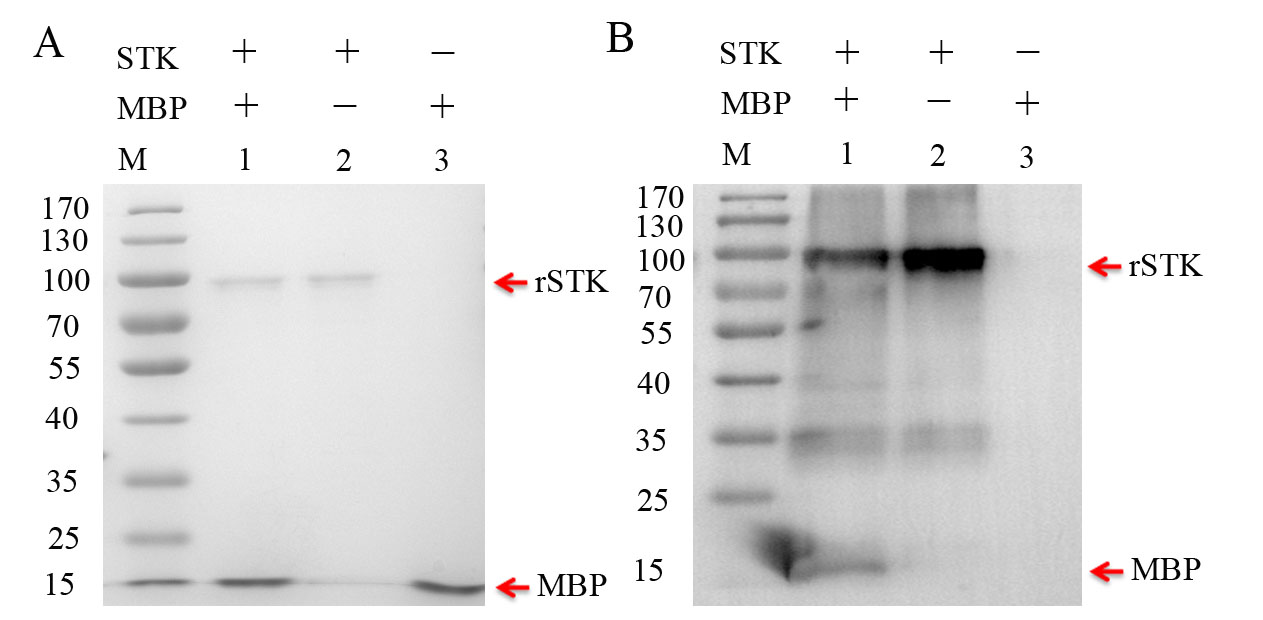

Supplement: Figure S2 — Kinase activity of STK in vitro. (A) After incubating rSTK with MBP as a positive substrate control, the protein was separated by SDS-PAGE and stained with Coomassie blue. (B) After incubating rSTK with MBP, the proteins were separated by SDS-PAGE, electroblotted, and then probed with an anti-pThr antibody. [file Image2.JPEG]

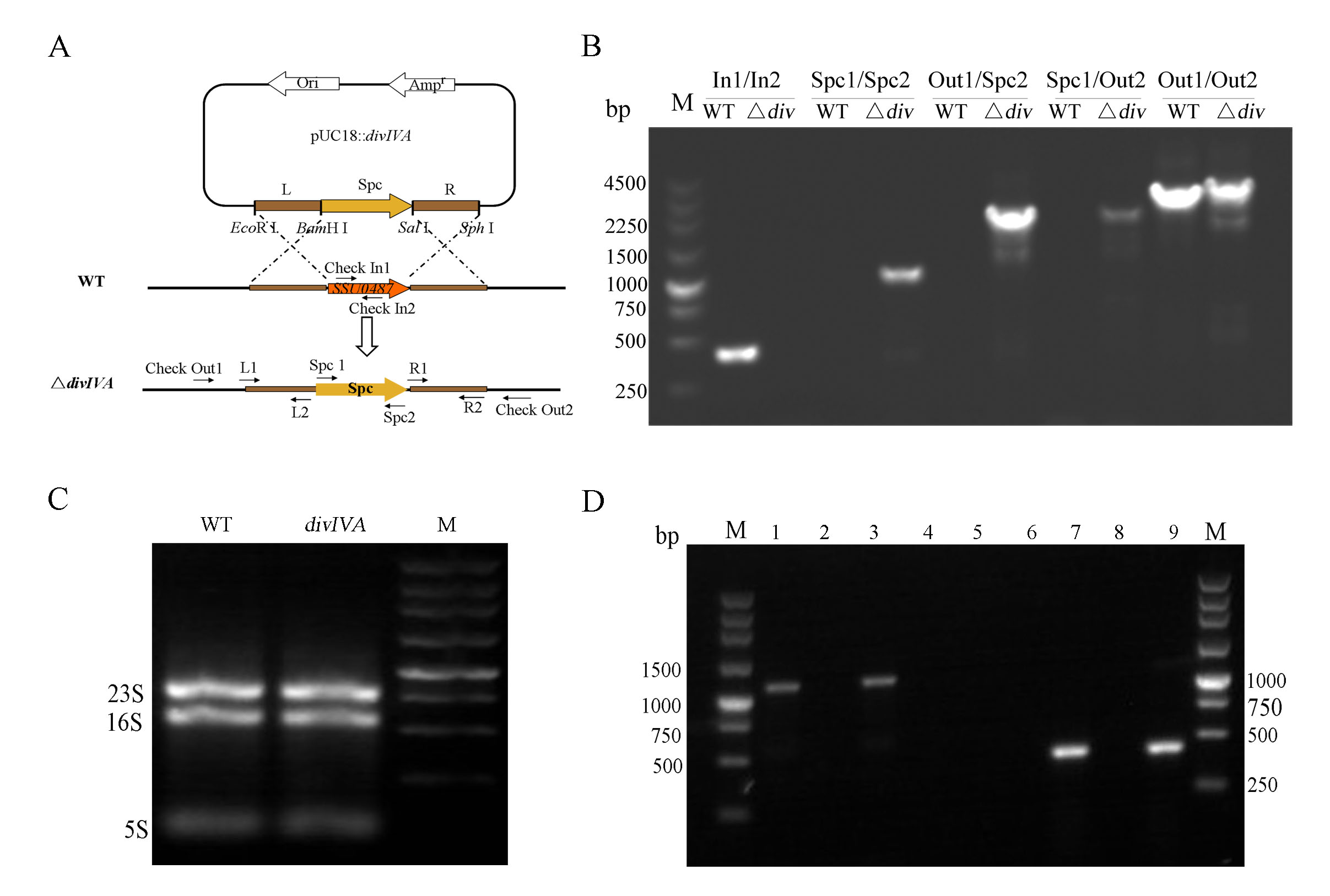

Supplement: Figure S3 — Construction of an isogenic divIVA mutant of S. suis 05ZYH33. (A) Diagram of gene divIVA knockout. The divIVA gene represented by a thick orange arrow was replaced by the spectinomycin resistance gene, as indicated by a yellow arrow. The thin arrows indicate the primer positions used in the construction and identification of the divIVA knockout mutant (ΔdivIVA). (B) Confirmatory PCRs of the ΔdivIVA mutant. The primer pairs and templates used in the PCR analysis are indicated above the lanes. The WT and Δdiv represent genome DNA of the wild-type strain 05ZYH33 and mutant strain, respectively. (C) Extraction of total RNAs. Total RNAs were extracted from the wild-type strain 05ZYH33 and the ΔdivIVA mutant. (D) RT-PCR analysis of the divIVA gene transcripts. The transcripts of the spc and divIVA genes were detected using reverse transcription (RT-PCR) analysis with cDNA, cDNA reaction mixtures without reverse transcriptase (cDNA-RT), or genomic DNA (gDNA) as templates. The RT-PCR products were analyzed by electrophoresis on a 1.0% agarose gel (lanes 1, 2, and 3 represent the application using gDNA, cDNA-RT, and cDNA from the mutant ΔdivIVA strain as template, respectively, and Spc1/Spc2 primers; lanes 4, 5, and 6 represent the application using gDNA, cDNA-RT, and cDNA from the mutant ΔdivIVA strain as a template, respectively, and In1/In2 primers; and lanes 7, 8, and 9 represent the application using gDNA, cDNA-RT, and cDNA from the wild-type strain 05ZYH33 as a template respectively, and In1/In2 primers). [file Image3.JPEG]
