# Supplementary material for: Streptococcus suis DivIVA Protein Is a Substrate of Ser/Thr Kinase STK and Involved in Cell Division Regulation
Source: Front Cell Infect Microbiol. 2018 Mar 20;8:85. doi: 10.3389/fcimb.2018.00085 (PMC5869912; doi:10.3389/fcimb.2018.00085)
Supplement: Table S2 — Bacterial strains and plasmids used in this study. [file Table2.DOC]

Table S2. Bacterial strains and plasmids used in this study.

| **Strains, plasmids or cell lines** | **Characteristics** | **Source** |
| --- | --- | --- |
| ***Streptococcus suis* 2** |  |  |
| 05ZYH33 | Virulent strain isolated from a dead patient with STSS | Laboratory collection |
| Δ*divIVA* | Isogenic Δ*divIVA* deletion mutant of strain 05ZYH33; Spc^r^ | This study |
| Δ*stk* | Isogenic Δ*stk* deletion mutant of strain 05ZYH33; Spc^r^ | Ref[59] |
| ***Escherichia coli*** |  |  |
| DH5α | Cloning host for recombinant plasmid | Transgen |
| TOP10 | The expression host of recombinant STK | Transgen |
| BL21 | The expression host of recombinant DivIVA | Transgen |
| **Plasmids** |  |  |
| pMD18-T | *E. coli* cloning vector, lacZ, Amp^r^ | Takara |
| pUC18 | *E. coli* cloning vector, lacZ, Amp^r^ | Takara |
| pUC18::*divIVA* | A recombinant vector of pUC18, designed for knock-out of *divIVA*, Amp^r^, Spc^r^ | This study |
| pSET2 | *E. coli*-*S. suis* shuttle vector; Spc^r^ | Takamatsu et al. |
| pET28a | T7 *lac* expression vector, His·Tag, Kan^R^ | Promega |
| pET28a::*divIVA* | Cloning expression of recombinant DivIVA | This study |
| pET30a | T7*lac* expression vector, His·Tag, Kan^R^ | Promega |
| pET30a::*stk* | Cloning expression of recombinant STK | This study |
